# Association of pleomorphic adenoma gene 1 with body weight and measurement of Bali cattle (*Bos javanicus*)

**DOI:** 10.14202/vetworld.2022.782-788

**Published:** 2022-03-30

**Authors:** Muhammad Cahyadi, Sukaryo Sukaryo, Mohammad Ilham Dhiaurridho, Thoriq Aldri Bramastya, Yuli Yanti, Joko Riyanto, Slamet Diah Volkandari, Pita Sudrajad

**Affiliations:** 1 Department of Animal Science, Faculty of Agriculture, Universitas Sebelas Maret, Surakarta, Indonesia; 2 Research Center for Biotechnology, Research Organization for Life Sciences, National Research and Innovation Agency (BRIN), Cibinong, Jawa Barat, Indonesia; 3 Assessment Institute for Agricultural Technology – Central Java, Indonesian Agency for Agricultural Research and Development, Ministry of Agriculture, Semarang, Indonesia

**Keywords:** association study, Bali cattle, body length, growth trait, Pleomorphic adenoma gene 1

## Abstract

**Background and Aim:**

Pleomorphic adenoma gene 1 (*PLAG1*) encodes a multifunctional transcription factor that controls many genes and pathways and is associated with cattle body weight and measurements. This study aimed to evaluate the association between *PLAG1* polymorphisms with body weight and measurements in Bali cattle.

**Materials and Methods:**

A total of 87 Bali cattle, consisting of 48 bulls and 39 heifers at the Breeding Center for Bali Cattle, were used as the population in this study. Cattle were 2 years old and kept semi-intensively in the pasture. Phenotype data consisting of body weight, withers height, body length, chest girth, waist height, and chest depth were measured. Birth weight data were obtained from birth records, and weight gain, adjusted weaning weight, and yearling weight were calculated using formulas. Blood samples were taken from the jugular vein as much as 5 mL, and genomic DNA was isolated using the salting-out method. Polymerase chain reaction (PCR) was performed to amplify three target polymorphisms, namely, g.48308 C>T, g.32212 (19 bp indel), and g.45233 T>C. The presence of a 19 bp indel was determined by direct observation of the PCR product on a 2% agarose gel. Two other polymorphisms were detected by PCR-restriction fragment length polymorphism using the restriction endonuclease enzymes *Sac*II and *Bcl*I. *PLAG1* genotype and phenotype associations were analyzed using a general linear model.

**Results:**

The results showed that two of the target polymorphisms in *PLAG1* did not vary. The DD genotype indicated by 123 bp of PCR product was the only genotype identified for g.32212 19 bp indel, and TT genotype was the only genotype found for g.45233 T>C single-nucleotide polymorphism (SNP). Conversely, g.48308 C>T SNP was found to be polymorphic. In addition, the g.48308 C>T polymorphism of *PLAG1* was significantly associated with body length of Bali cattle. Cattle with the CC genotype had a greater body length than the other two genotypes.

**Conclusion:**

The g.48308 C>T SNP in *PLAG1* was associated with Bali cattle body length characteristics. This finding could be used as a basis for selecting Bali cattle based on body length characteristics.

## Introduction

Bali cattle (*Bos javanicus*) are indigenous Indonesian cattle, which are hypothesized to have originated from the domestication of wild *Banteng* long ago. Bali cattle breed well on Bali Island because the Balinese culture venerates cattle [1]. In 1986, the Government of Indonesia established the Pulukan Breeding Center which is now known as the Balai Pembibitan Ternak Unggul dan Hijauan Pakan Ternak (BPTU-HPT) Denpasar. It was founded 10 years after the Bali cattle breeding and development project started. The project focused on the improvement of Bali cattle population and its genetic quality by conventional breeding program [1]. Bali cattle have several advantageous characteristics, including good adaptability to tropical climates, high fertility, resistance to parasites, and low meat fat content [2,3]. Therefore, Bali cattle are one of the national cattle genetic resources that need to be maintained and used sustainably to optimize these advantages. Bali cattle are also listed as a cattle breed by the Food and Agriculture Organization [4].

Bali cattle are a cattle breed with the largest population in Indonesia, reaching 32.3% [5]. Therefore, Bali cattle play an important role in satisfying national meat consumption of 2.31 kg per capita, equating to the need for 624,162 tons of red meat [6]. However, this high demand is not supported by the amount of meat produced, which only reached 515,600 tons in 2020 [7]. The low production of meat could be overcome by utilizing the potential of Bali cattle as a native Indonesian genetic resource. Improving the genetic quality of Bali cattle must be conducted continuously and in a planned manner with measurable milestones. One way to increase the productivity of Bali cattle is through marker-assisted selection (MAS). The development of molecular genetics has enabled the identification of multiple genes and genetic markers associated with genes responsible for desired phenotypic traits, including quantitative trait loci (QTL) or genomic regions affecting quantitative traits and genes for a particular trait [8,9]. A widely reported gene affecting livestock productivity is the pleomorphic adenoma gene 1 (*PLAG1*) gene [10].

*PLAG1* is a member of the pleomorphic adenoma gene family along with *PLAGL1* and *PLAGL2*, which express a class of zinc-finger proteins [11]. The *PLAG1* encodes a multifunctional transcription factor that controls many genes and pathways, such as the Insulin-like growth factor (IGF)-II, IGF-1R, and WNT pathways [12]. Previous studies have reported that *PLAG1* affects the stature and body weight of dairy and beef cattle [13,14]. Hartati *et al*. [10] found that a single-nucleotide polymorphism (SNP) in *PLAG1* was positively associated with Indonesian Peranakan Ongole cattle body measurements. *PLAG1* plays a role in controlling the increase in body measurements and height in Japanese Black cattle [15]. In addition to body measurements, polymorphisms in *PLAG1* also affect cattle body weight and reproductive characteristics [16]. Xu *et al*. [17] reported that a 19 bp indel in *PLAG1* was associated with growth traits and body measurements in Pinan, Xianan, and Jiaxian cattle in China. Zhong *et al*. [18] reported that the g.48308C>T polymorphism of *PLAG1* significantly affected height and chest girth in five Chinese cattle breeds, and individuals with the CC genotype were preferred for these traits.

Few studies have evaluated *PLAG1* in *B. javanicus* because of the limited characteristics of uniform cattle in a large population [19,20]. This study aimed to evaluate the association between *PLAG1* polymorphisms with body weight and measurements in Bali cattle.

## Materials and Methods

### Ethical approval

All animal procedures related to samples of Bali cattle were approved by the Ethical Clearance Commission, National Research and Innovation Agency (BRIN) No. 81/Klirens/X/2021.

### Study period and location

The study was conducted from March to November 2021. Blood samples of Bali cattle were collected at the Breeding Center of Bali Cattle (Denpasar, Indonesia). The laboratory works were carried out at the Division of Biology, Integrated Laboratory of Universitas Sebelas Maret.

### Bali cattle population and measurement of phenotype data

Bali cattle used in this study were raised in the Bali cattle breeding center located at Pangyangan, Pekutatan, Jembrana Regency, Bali. The breeding center is located at an altitude of 125 m above sea level. It has an average rainfall of 485 mm/month with a temperature ranging from 22 to 30°C and average relative humidity of 70%.

A total of 87 Bali cattle, consisting of 48 bulls and 39 heifers, were obtained from two male paddocks and two female paddocks. The average age of the cattle was 2 years, and they were maintained semi-intensively with complete records. Up to 4 kg/animal of additional feed was given in the form of concentrate in the morning. Although cattle easily received forage from the paddock, additional king grass was given twice in the morning and afternoon, at a rate of 15 kg/animal. Drinking water was provided *ad libitum*. This management was the same for all cattle kept in the paddock. All cattle in this study had been vaccinated for *Septicemia epizootica* and Jembrana diseases.

**Figure-1:**
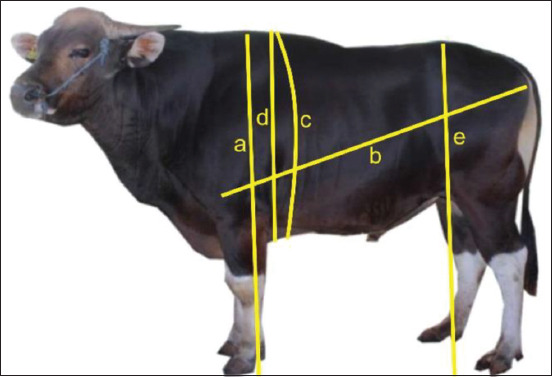
Body measurement of Bali cattle according to SNI 7651-4: 2017. Line (a) represents withers height; (b) body length; (c) chest girth; (d) chest depth; and (e) waist height.


Body weight data were measured using a Tru-Test EziWeigh7i digital scale (Datamars, Auckland, New Zealand) and expressed in kilograms. Body measurement data consisting of withers height (a), body length (b), chest girth (c), chest depth (d), and waist height (e) were measured using a ruler and measuring tape following SNI [21] and were calculated in centimeter (Figure-1). Birth weight data were obtained from records at BPTU-HPT Bali Cattle. Furthermore, data on weight gain, weaning weight, and yearling weight were calculated using the formulas reported by Chenette and Frahm [22] as follows:


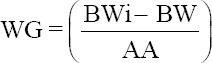


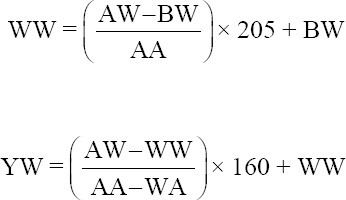


Where, WG is weight gain, BWi is actual body weight, BW is birth weight, WW is adjusted 205 d weaning weight, AW is actual body weight, YW is adjusted 365 days yearling weight, AA is actual age (d), and WA is weaning age. Body weight was expressed in kilograms, and age was expressed in days.

### Blood sample collection and DNA extraction

Blood samples were collected from the jugular vein of the cattle as much as 5 mL using an 18G vacuum needle in a 10 mL Vacutainer tube containing EDTA. The collected blood samples were stored in the cooling box at 0°C during transportation and kept in the refrigerator at 4°C until further analysis. DNA extraction from blood samples was conducted following the high salt method protocol by Montgomery and Sise [23]. The genomic DNA obtained was quantified using a NanoPhotometer (P-Class^®^, Implen, Munich, Germany); DNA concentration (ng/μL) and DNA purity were obtained by comparing the optical density at 260 and 280 nm. The concentration of genomic DNA in this study was set at a minimum of 20 ng/μL and purity greater than 1.8.

### Amplification and genotyping of PLAG1

The amplification of *PLAG1* targeted three polymorphisms, namely, g.48308 C>T, g.32212 (19 bp indel), and g.45233 T>C. Polymerase chain reaction (PCR) was conducted using a MiniAmp^®^ Thermal cycler machine (Thermo Fisher Scientific, Singapore). The PCR reaction consisted of 12.5 μL GoTaq(R) Green Master Mix (Promega, Madison, USA), 9.5 μL nuclease-free water (1^st^ BASE, Singapore), 1 μL of each primer (Table-1) [17,18], and 1 μL of template DNA. All materials were mixed in a PCR tube with a total volume of 25 μL. The amplification of *PLAG1* was initiated by pre-denaturation at 95°C for 5 min, followed by 35 cycles of denaturation at 95°C for 30 s, annealing, and extension at 72°C for 30 s, and the reaction was completed after a final extension at 72°C for 10 min. The annealing temperature and time are listed in Table-1. The PCR products were run on a 2% agarose gel stained with ethidium bromide (Promega) using the Submarine Electrophoresis System Mufid ex (Advance, Tokyo, Japan) for 30 min at 110 V. The 100 bp marker ladder (Geneaid, Taiwan) was used as the standard for the DNA band size. The agarose gel was then visualized using a Gel Documentation System (Glite UV, Pacific Image, Taiwan).

**Table-1:** Primer pairs of pleomorphic adenoma gene 1 used in this study.

Polymorphism	Primer (5’ to 3’)	Annealing (°C/s)	Amplicon (bp)	RE	Reference
g. 48308 C>T	F: gcgcgtatcagtcaggacat	58/45	628	*Sac*II	[18]
	R: cctttgcctgttgctttccc				
g. 32212 19 bp indel	F: tccgaacaacaggtgagggagaaat	60/30	142/123	-	[17]
	R: ccacttcaggggtgctctaggtttg				
g. 45233 T>C	F: gcgtgaaggagaagaagcac	56/45	767	*Bcl*I	This study
	R: gatcgggttatagggagggc				

RE = Restriction enzyme

Genotyping was conducted by observing DNA bands of PCR products to detect the 19 bp indel, and digestion of PCR products was conducted using the PCR-restriction fragment length polymorphism (PCR-RFLP) technique to detect other polymorphisms. PCR-RFLP was performed according to the FastDigest *Sac*II protocol for the g.48308 C>T SNP and Fast Digest *Bcl*I for the g.45233 T>C SNP (Thermo Fisher Scientific Inc., Vilnius, Lithuania). The individual Bali cattle genotype was determined by 2% agarose gel electrophoresis of the digested PCR product. The gel was then visualized under UV light using a Gel Documentation System. PCR products of three DNA pools were sequenced to confirm amplicon size (Apical Scientific, Malaysia).

### Statistical analysis

Genotype and allele frequencies were calculated, and Pearson’s Chi-square test was conducted to verify the Hardy-Weinberg equilibrium (HWE) status. A general linear model was applied to evaluate the effects of *PLAG1* polymorphisms on body weight and measurements using MINITAB version 14.0 software (Minitab Inc., USA). Association analysis was performed using the following model:

Y_ijk_=μ+G_i_+S_j_+ɛ_ijk_

Where, Y_ijk_ is the phenotype of the k^th^ animal, μ is the population mean, Gi is the fixed effect of genotype, Sj is the fixed effect of sex, and ɛ_ijk_ is the residual error associated with the k^th^ animal. Tukey’s test was performed to determine pairwise differences among the genotypes.

## Results

### Amplification and genotyping of *PLAG1*

**Figure-2:**
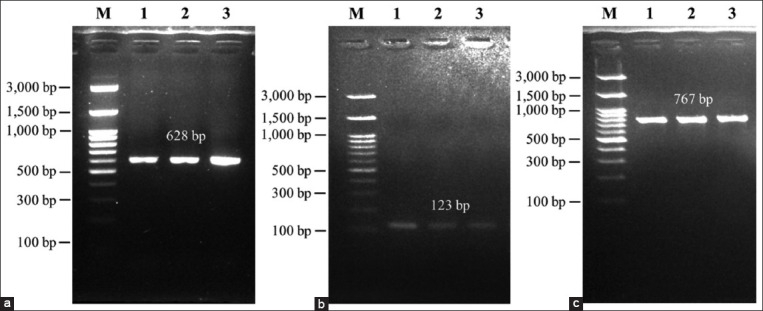
Amplification of pleomorphic adenoma gene 1 polymorphisms. (a) Is an amplicon for detecting the g.48308 C>T single-nucleotide polymorphism (SNP), (b) for detecting the 19 bp indel, and (c) for detecting the g.45233 T>C SNP.

**Figure-3:**
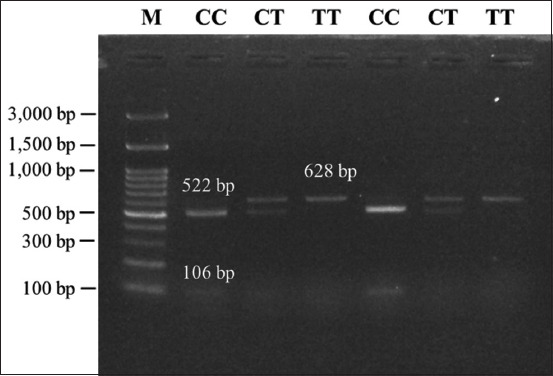
Genotyping of the pleomorphic adenoma gene 1 based on the g.48308 C>T single-nucleotide polymorphism. M is the 100 bp marker ladder, CC represents Bali cattle with the CC genotype, CT represents Bali cattle with the CT genotype, and TT represents Bali cattle with the TT genotype.

Three DNA fragments specific to *PLAG1*, 628 bp, 123 bp, and 767 bp, were successfully amplified using PCR (Figure-2). The 628 bp DNA fragment represented the g.48308 C>T SNP located in the 3’UTR region and was recognized by *Sac*II. There were three genotypes, namely, the CC (502 bp and 126 bp), TC (628 bp, 502 bp, and 126 bp), and TT genotypes (628 bp, the fragment could not be digested by the restriction enzyme). Thus, this study identified three genotypes in the Bali cattle population (Figure-3).

A 19 bp indel is located in intron 3, specifically at nucleotide number 32212, of *PLAG1*. This polymorphism was detected by the presence of a 19 bp indel, giving rise to three types of genotypes, namely, II, ID, and DD. Genotype II was characterized by the appearance of one band at 142 bp and the DD genotype at 123 bp, whereas the heterozygous ID produced two bands, 142 bp and 123 bp. The genotype detected in the Bali cattle population was the monomorphic DD genotype (100%). Another SNP identified in *PLAG1*, namely, the g.45233 T>C SNP, located in exon 4, is a missense variant that has been successfully amplified in a 767 bp DNA fragment. However, this SNP did not vary in the Bali cattle population.

### Allele and genotype frequencies of *PLAG1* in Bali cattle

The analysis showed that the CC genotype was dominant (88.5%), with an allele frequency of 0.925 (Table-2). Furthermore, based on the HWE analysis, the Bali cattle population experienced an imbalance, as indicated by the HWE value of 15.21, which was greater than the X^2^ value with p<0.05. Furthermore, the 19 bp indel and g.45233 T>C SNP were monomorphic. All individual Bali cattle genotypes were homozygous (DD and TT) for both polymorphisms. Thus, they could not be used for the association analyses.

**Table-2:** Allele and genotype frequencies of the g.48308 C>T single-nucleotide polymorphism of pleomorphic adenoma gene 1.

Genotype frequency			Allele frequency		χ^2^	p-value
	
CC	CT	TT	C	T		
0.8850	0.0805	0.0345	0.925	0.075	15.21	9.6429E-05

χ*^2^*=Chi-square

### Association of *PLAG1* polymorphisms with body weight and traits

Analysis of the associations between the g.48308 C>T SNP and body size and weight in Bali cattle are shown in Table-3. Statistically, the SNP g.48308 C>T was significantly associated with body length in the Bali cattle population. Individuals with the CC genotype had a greater body length than those with the other genotypes (p=0.004). In addition, these SNPs tended to be significantly associated with waist height and chest depth in Bali cattle (p=0.066 and p=0.067, respectively).

**Table-3:** Association between the g.48308 C>T single-nucleotide polymorphism of pleomorphic adenoma gene 1 and body weight and traits in Bali cattle.

Trait	Mean±SE			p-value

	CC (n=77)	CT (n=7)	TT (n=3)	
Birth weight (kg)	18.94±0.28	18.86±0.71	19.00±1.00	0.794
Weaning weight (kg)	72.03±2.66	67.66±4.30	74.76±1.06	0.103
Yearling weight (kg)	113.47±4.64	105.75±7.45	118.27±2.27	0.101
Body weight (kg)	214.34±8.22	209.00±13.80	187.00±18.60	0.069^†^
Weight gain (kg/day)	0.26±0.01	0.24±0.02	0.27±0.01	0.101
Withers height (cm)	112.19±0.69	110.14±2.37	109.67±3.33	0.142
Body length (cm)	107.52^a^±1.17	101.86^b^±2.65	103.67^b^±1.86	0.004**
Chest girth (cm)	147.04±1.72	147.71±3.33	140.00±4.04	0.109
Waist height (cm)	111.86±0.67	107.86±2.65	109.50±1.50	0.066^†^
Chest depth (cm)	57.468±0.71	55.57±1.73	55.50±2.50	0.067^†^

SE is standard error; n is number of samples

** indicates highly significant effect

† indicates a suggestive-significance effect.

## Discussion

*PLAG1* is a proto-oncogene that encodes a zinc-finger containing transcription factor and is involved in many pathways. It is located on bovine chromosome 14 (BTA14). Based on the ensemble database, *PLAG1* spans nucleotides 23,330,541-23,375,751 bp on BTA14 and has five exons, four introns, and five transcripts [24]. This study is the first to determine an association between the g.48308 C>T SNP of *PLAG1* and body length in a Bali cattle population. Individuals with the CC genotype possess a greater body length than cattle with other genotypes. This polymorphism has previously been reported to be associated with growth traits in five breeds of cattle in China, revealing that cattle with the CC genotype had greater height and chest girth than cattle with the TT genotype [18]. However, the analysis showed that the Bali cattle population in this study was not in HWE. This can be caused by migration, mutation, recombination, or selection efforts in the population [25]. The Bali cattle population used in this study was obtained from BPTU-HPT Bali cattle, which are allotted the task of providing superior Bali cattle. A selection process may have been employed by the agency considering that its task is to maintain and provide superior Bali cattle breeds for the community. In addition, Bali cattle breed well on Bali Island because Balinese culture respects cattle and this culture is not found in other parts of Indonesia [1,3,4]. This study also identified two other polymorphisms, namely, 19 bp indel and SNP g.45233 T>C, but these two polymorphisms were monomorphic and could not be associated with body weight and measurement traits. Previous research conducted by Xu *et al*. [17] who showed a positive association between the 19 bp indel in *PLAG1* and growth traits in Pinan, Xianan, and Jiaxian cattle in China. Different results were reported by Peng *et al*. [26] who found that the 19 bp indel in *PLAG1* was not associated with phenotypic traits of Xianjian brown, red steppe, and Yunling cattle. *PLAG1* has been widely reported to be associated with growth traits, body size or stature, and reproductive traits in various breeds of cattle [10,13-18]. It affects hip height, growth rate, carcass weight, body condition score, birth weight, and weight at different stages of age [27-30]. It is also responsible for growth physiology [31] in milk production, body size, coat color, and muscle formation in cattle [32]. The association between birth weight and *PLAG1* genotype has been verified in a Friesian Holstein dairy cattle population. The results showed that *PLAG1* is related to body size regulation [33]. This statement is supported by Abi Habib *et al*. [31] who reported that *PLAG1* plays a role in growth physiology. Functional mutations in the bovine *PLAG1* have also been reported to be associated with stature in beef cattle [12]. In addition, an epistatic interaction of the *PLAG1* polymorphism with other genes, such as *IGF2* and insulin, has been reported in cattle [34]. QTL regions that significantly affect livestock height have been mapped to a region on chromosome 14. The mapping of quantitative trait nucleotides (QTNs) to the *PLAG1*-*CHCHD7* intergenic region shows a positive association with cattle body size [13]. Moreover, a pleiotropic QTN named bovine HD1400007259 in the *PLAG1*-*CHCHD7* gene region of BTA14 was shown to be significantly associated with bicep and calf muscle size [35].

Selection based on genetic markers of growth traits is very effective in improving cattle performance. Bali cattle, indigenous Indonesian beef cattle, must be given more attention, and productivity must be increased through MAS [36]. Genetic marker-based livestock selection programs have been very effective because they can be conducted as early as needed; thus, they are more efficient than conventional livestock selection [37]. In addition, growth traits have a heritability of up to 0.43, which means that 43% of growth traits are affected by genetics [38]. The nature of growth is represented by body weight and measurements of livestock. This study showed that *PLAG1* is one of the candidate genes responsible for growth traits of Bali cattle since g.48308 C>T SNP was significantly affected body length of Bali cattle. Therefore, the results of this study could be used as a basis for developing policies to improve the genetic quality of Bali cattle by the government through the BPTU-HPT Bali Cattle. Consequently, the function of the Bali Cattle Breeding Center as a provider of superior Bali cattle breeds could be optimized.

## Conclusion

The g.48308 C>T SNP of *PLAG1* was associated with the body length trait of Bali cattle. This finding could be used as a foundation for selecting Bali cattle based on body measurement characteristics using MAS, which is much more effective and efficient than conventional phenotypic selection. However, validation in different populations with a larger number of Bali cattle should be taken into account to achieve more reliable results.

## Authors’ Contributions

MC: Conceptualization, methodology, formal analysis, funding acquisition, supervision, and writing – review and editing. SS: Validation, investigation, data curation, writing – original draft preparation. MID: Validation, writing – original draft, and project administration. TAB: Validation and writing – original draft. YY and JR: Resources and writing – review and editing. SDV and PS: Investigation and writing – original draft. All authors read and approved the final manuscript.

## References

[1] Talib C (2002). Sapi Bali di daerah sumber bibit dan peluang pengembangannya [Bali cattle in breeding areas and opportunities for their development]. Wartazoa.

[2] Gunawan I.W, Suwiti N.K, Sampurna P (2016). Pengaruh pemberian mineral terhadap lingkar dada, panjang dan tinggi tubuh sapi bali jantan [The effects of minerals on the chest circumference, body length and body height of male Bali cattle]. Bul. Vet. Udayana.

[3] Warmadewi D.A, Oka I.G.L, Ardika I.N (2017). Efektivitas seleksi dimensi tubuh sapi Bali induk [The effectiveness of selection of the body dimensions of Bali cow]. Majalah Ilmu Peternakan.

[4] Hikmawaty H, Bellavista B, Mahmud A.T.B, Salam A (2018). Korelasi bobot badan dan variabel-variabel ukuran tubuh sebagai dasar seleksi calon induk sapi Bali [Correlation of body weight and body sizes as the basis for selection of prospective Bali cows]. Agrovital J. Ilmu Pertanian.

[5] Badan Pusat Statistik. Pendataan Sapi Potong, Sapi Perah, dan Kerbau 2011 (Data Collection of Beef and Dairy Cattle, and Buffalo 2011) (2011). Badan Pusat Statistik, Jakarta.

[6] Pusat Data dan Sistem Informasi Pertanian. Outlook Daging sapi (Outlook of Beef) (2020). Sekretariat Jenderal Kementerian Pertanian, Jakarta.

[7] Direktorat Jenderal Peternakan dan Kesehatan Hewan. Statistik Peternakan dan Kesehatan Hewan (Statistics of Livestock and Animal Health) (2020). Direktorat Jenderal Peternakan dan Kesehatan Hewan, Kementerian Pertanian RI, Jakarta.

[8] Dekkers J.C.M (2004). Commercial application of marker-and gene-assisted selection in livestock:Strategies and lessons. J. Anim. Sci.

[9] Williams J.L (2005). The use of marker-assisted selection in animal breeding and biotechnology. Rev. Sci. Tech.

[10] Hartati H, Utsunomiya Y.T, Sonstegard T.S, Garcia J.F, Jakaria J, Muladno M (2015). Evidence of *Bos javanicus* x *Bos indicus* hybridization and major QTLs for birth weight in Indonesian Peranakan Ongole cattle. BMC Genet.

[11] Kas K, Voz M.I, Roijer E, Astrom A.K, Mayen E, Stenam G, Van de Ven W.J.M (1997). Promoter swapping between the genes for a novel zinc finger protein and beta-casein in pleiomorphic adenomas with t (3;8) (p21;q12) translocations. Nat. Genet.

[12] Fortes M.R.S, Reverter A, Kelly M, McCulloch R, Lehnert A (2013). Genome-wide association study for inhibin, luteinizing hormone, insulin-like growth factor 1, testicular size and semen traits in bovine species. Andrology.

[13] Karim L, Takeda H, Lin L, Druet T, Arias J.A.C, Baurain D, Cambisano N, Davis S.R, Farnir F, Grisart B, Harris B.L, Keehan M.D, Littlejohn M.D, Spelman R.J, Georges M, Coppieters W (2011). Variants modulating the expression of a chromosome domain encompassing PLAG1 influence bovine stature. Nat. Genet.

[14] Fortes M.R.S, Kemper K, Sasazaki S, Reverter A, Pryce J.E, Barendse W, Bunch R, McCulloch R, Harrison B, Bolormaa S, Zhang Y.D, Hawken R.J, Goddard M.E, Lehnert S.A (2013). Evidence for pleiotropism and recent selection in the PLAG1 region in Australian Beef cattle. Anim. Genet.

[15] Takasuga A (2016). PLAG1 and NCAPG-LCORL in livestock. J. Anim. Sci.

[16] Utsunomiya Y.T, Milanesi M, Utsunomiya A.T.H, Torrecilha R.B.P, Kim E.S, Costa M.S, Aguiar T.S, Schroeder S, Carmo A.S, Carvalheiro R, Neves H.H.R, Padula R.C.M, Sussai T.S, Zavarez L.B, Cipriano R.S, Caminhas M.M.T, Hambrecht G, Eufemi L.C.E, Marsan P.A, Cesana D, Sannazaro M, Buora M, Morgante M, Liu G, Bickhart D, Tassell C.P.V, Sölkner J, Sonstegard T.S, Garcia J.F (2017). A PLAG1 mutation contributed to stature recovery in modern cattle. Sci. Rep.

[17] Xu W, He H, Zheng L, Xu J.W, Lei C.H, Zhang G.M, Dang R.H, Nui H, Qi X.L, Chen H, Huang Y.Z (2018). Detection of 19-bp deletion within PLAG1 gene and its effect on growth traits in cattle. Gene.

[18] Zhong J.L, Xu J.W, Wang J, Wen Y.F, Niu H, Zheng L, He H, Peng K, He P, Shi S.Y, Huang Y.Q, Lei C.Z, Dang R.H, Lan X.Y, Qi X.L, Chen H, Huang Y.Z (2019). A novel SNP of PLAG1 gene and its association with growth traits in Chinese cattle. Gene.

[19] Putra I.G.R, Sari D.A, Rachmawati S.M, Oktaviani R, Noor R.R, Jakaria J (2021). Identification of single nucleotide polymorphism c.957A>C of PLAG1 gene and its association with growth traits in Bali cattle. J. Indones. Trop. Anim. Agric.

[20] Sukaryo S, Augustin R, Yanti Y, Riyanto J, Volkandari S.D, Sudrajad P, Cahyadi M (2022). Identification of 19-bp indel of the Pleomorphic adenoma gene 1 in Bali cattle population. E3S Web Conf.

[21] Standar Nasional Indonesia (2017). Bibit Sapi Potong-bagian 4 (Superior Beef Calves-part 4).

[22] Chenette C.G, Frahm R.R (1981). A comparison of different age-of dam and sex correction factors for birth, weaning, and yearling weights in beef cattle. Anim. Sci. Res. Rep.

[23] Montgomery G.W, Sise J.A (1990). Extraction DNA from sheep white blood cells. J. Agric. Res.

[24] Hou J, Qu K, Jia P, Hanif Q, Zhang J, Chen N, Dang R, Chen H, Huang B (2020). A SNP in PLAG1 is associated with body height trait in Chinese cattle. Anim. Genet.

[25] Alternberg L, Liberman U, Feldman M.W (2017). Unified reduction principle for the evolution of mutation, migration, and recombination. Proc. Natl. Acad. Sci.

[26] Peng K, Zhang G.L, Yu T, Cao Y, Yu Y.S, Chen H, Lei C.Z, Lan X.Y, Zhao Y.M (2020). Detection of Indel variations within seven candidate genes and their associations with phenotypic traits in three cattle breeds. Anim. Biotechnol.

[27] Nishimura S, Watanabe T, Mizoshita K, Tatsuda K, Fujita T, Watanabe N, Sugimoto Y, Takasuga (2012). Genome-wide association study identified three major QTL for carcass weight, including the PLAG1-CHCHD7 QTN for stature in Japanese Black cattle. BMC Genet.

[28] Hawken R.J, Zhang Y.D, Fortes M.R.S, Colis E, Barris W.C, Corbet N.J, Williams P.J, Fordyce G, Holroyd R.G, Walkley J.R, Barendse W, Johnston D.J, Prayaga K.C, Tier B, Reverter A, Lenhert S.A (2012). Genome-wide association studies of female reproduction in tropically adapted beef cattle. Anim. Sci. J.

[29] Pitetti J.L, Calvel P, Zimmermann C, Conne B, Papaioannou M.D, Aubry F, Cederroth C.R, Urner F, Fumel B, Crausaz M, Docquier M, Herrera P.L, Pralong F, Germond M, Guillou F, Jégou B, Nef S (2013). An essential role for insulin and IGF1 receptors in regulating sertoli cell proliferation, testis size, and FSH action in mice. Mol. Endocrinol.

[30] Utsunomiya Y.T, do Carmo A.S, Carvalheiro R, Neves H.H.R, Matos M.C, Zavarez L.B, O'Brien A.M.P, Sölkner J, McEwan J.C, Cole J.B, Tassell C.P.V, Schenkel F.S, da Silva M.V.G.B, Neto L.R.P, Sonstegard T.S, Garcia J.F (2013). Genome-wide association study for birth weight in Nellore cattle points to previously described orthologous genes affecting human and bovine height. BMC Genet.

[31] Abi Habib W, Brioude F, Edouard T, Bennett J.T, Lienhardt-Roussie L, Tixier F, Salem J, Yuen T, Azii S, Le Bouc Y, Harbison M.D, Netchine I (2018). Genetic disruption of the oncogenic HMGA2-PLAG1-IGF2 pathway causes fetal growth restriction. Genet. Med.

[32] Zhao F, McParland S, Kearney F, Du L, Berry D.P (2015). Detection of selection signatures in dairy and beef cattle using high-density genomic information. Genet. Sel. Evol.

[33] Littlejohn M, Grala T, Sanders K, Walker C, Waghorn G, Macdonald K, Coppieters W, Georges M, Spelman R, Hillerton E, Davis S, Snell R (2012). Genetic variation in PLAG1 associates with early life body weight and peripubertal weight and growth in Bos Taurus. Anim. Genet.

[34] Bolormaa S, Pryce J.E, Zhang Y, Reverter A, Barendse W, Hayes B.J, Goddard M.E (2015). Non-additive genetic variation in growth, carcass and fertility traits of beef cattle. Genet. Sel. Evol.

[35] Song Y, Xu L, Chen Y (2016). Genome-wide association study reveals the PLAG1 gene for knuckle, biceps and shank weight in Simmental beef cattle. PLoS One.

[36] Sudrajad P, Volkandari S.D, Cahyadi M, Prasetyo A, Komalawati K, Wibowo S, Subiharta S (2021). Pemanfaatan informasi genom untuk eksplorasi struktur genetik dan asosiasinya dengan performan ternak di Indonesia (Utilization of genomic data to explore genetic structure and its association with performance of livestock in Indonesia). Livest. Anim. Res.

[37] Hayes B, Goddard M (2010). Genome-wide association and genomic selection in animal breeding. Genome.

[38] Gunawan A, Jakaria (2011). Genetic and non-genetic effect on birth, weaning, and yearling weight of Bali cattle. Med. Peternakan.

